# Two unrelated patients with autosomal dominant omodysplasia and *FRIZZLED2* mutations

**DOI:** 10.1002/ccr3.1818

**Published:** 2018-10-15

**Authors:** Hannah E. Warren, Raymond J. Louie, Michael J. Friez, Jaime L. Frías, Jules G. Leroy, Jürgen W. Spranger, Steven A. Skinner, Neena L. Champaigne

**Affiliations:** ^1^ Greenwood Genetic Center Greenwood South Carolina; ^2^ Department of Pediatrics University of South Florida Tampa Florida

**Keywords:** FRIZZLED2, omodysplasia, rhizomelia

## Abstract

Presented are two patients with autosomal dominant omodysplasia and mutations in the *FZD2* gene. The mutations identified have been recently reported, suggesting the possibility of recurrent mutations. The phenotypes of these patients overlap with what has been previously reported, though intellectual disability as seen in our patient is not typical.

## Graphical abstract

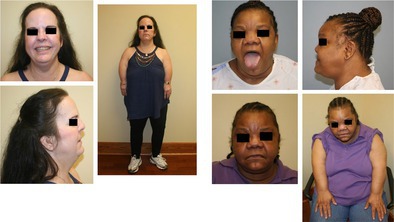

## 1 INTRODUCTION

Autosomal dominant omodysplasia (OMOD2) is a rare skeletal dysplasia delineated and clinically differentiated from its autosomal recessive counterpart (OMOD1) by Maroteaux et al.^1^ OMOD2 is clinically characterized primarily by short upper extremities, with rhizomelic greater than mesomelic shortness, radial dislocation, short first metacarpals, facial dysmorphism, and genital anomalies. Additional features that have been variably reported include short stature, femoral anomalies, and vertebral anomalies.

Saal et al^2^ recently described an alteration (c.1644G>A, p.Trp548*) in the *FRIZZLED2* (*FZD2*) gene causative of OMOD2. Subsequently, there have been two additional reports of a novel missense alteration (c.1301G>T, p.Gly434Val) and a novel nonsense alteration (c.1640C>A, p.Ser547*) in the *FZD2* gene associated with OMOD2.^3^, ^4^

The purposes of this report are to communicate the detection of the p.Trp548* *FZD2* alteration in a previously described patient and to describe a new unrelated patient initially considered to have features more consistent with Robinow syndrome.

## 2 CLINICAL REPORTS

### 2.1 Patient 1

**Figure 1:**
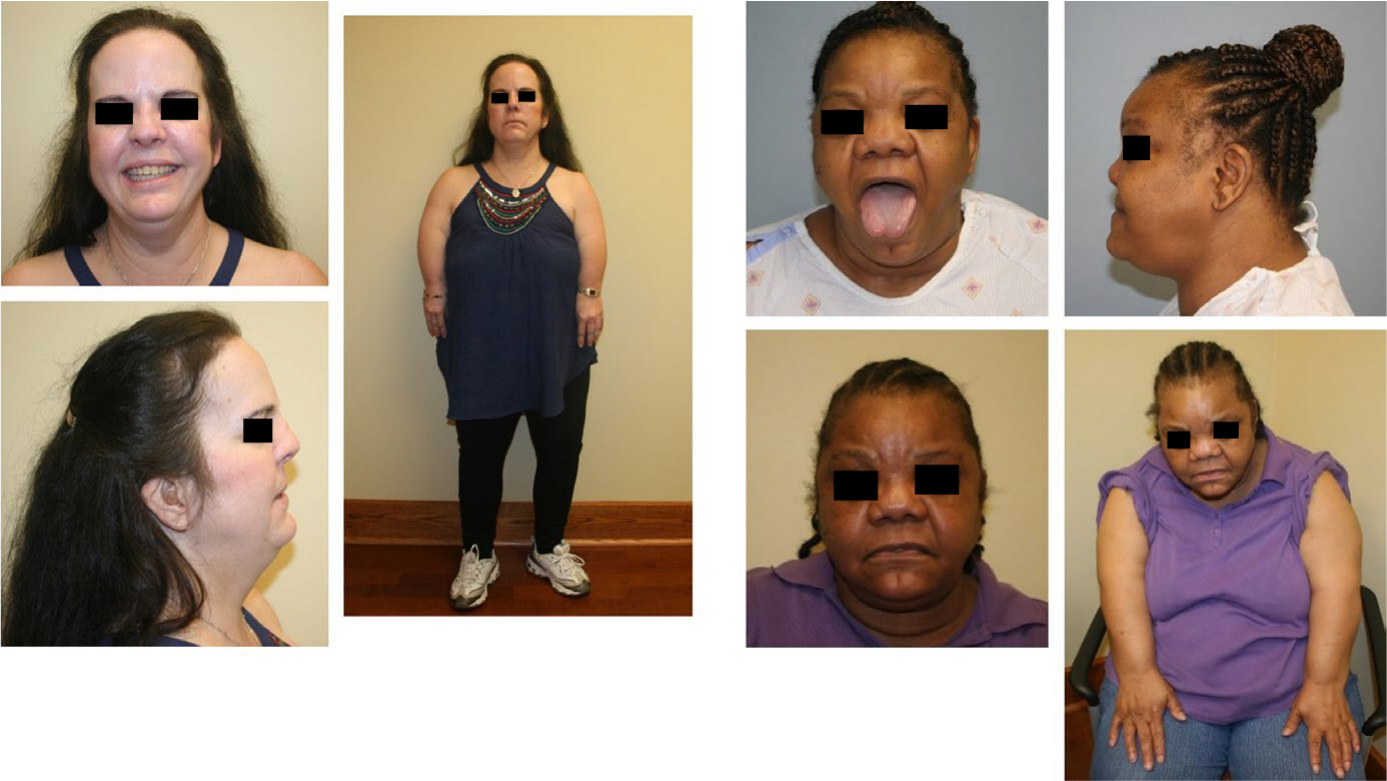
Patient 1 at age 52 years (left) and Patient 2 at 55 years (right top) and 63 years (right bottom). Note shortened upper extremities in both patients. Facial features of Patient 2 included a broad forehead, flat midface, broad nose with flat tip, anteverted nares, thick alae nasi, broad mouth, full lips, and broad chin. Palpebral fissures were narrow, upslanting, and hyperteloric. Interpupillary distance at age 57 years was measured at 7.5 cm (>97th centile). Subtle signs of midline clefting were noted including vertical furrowing in the glabellar region, a cleft in the chin, and a bifid tongue

Patient 1 is a Caucasian female depicted in Figure 1 who is currently 55 years of age. A detailed clinical report of her history was published prior to the identification of *FZD2* alterations as the cause of OMOD2.^5^ Clinical follow‐up confirmed her adequate general health, normal intellect, radiographic features, and provided the opportunity to detect the p.Trp548* *FZD2* alteration. This alteration was not identified in her unaffected mother and daughter, supporting its pathogenicity. It is identical to the alteration identified in the affected mother‐daughter pair presented by Saal et al.^2^

### 2.2 Patient 2

Patient 2 is a 65‐year‐old African‐American female, also depicted in Figure 1. She had an apparently normal perinatal and neonatal history, slow physical growth, and some learning difficulty in childhood. The patient began developing staring spells at approximately 6 years of age, which were eventually interpreted and treated as seizures. Pubertal development was apparently normal with menses starting at age 11. There was a report of heavy menses, and a hysterectomy was ultimately performed due to uterine fibroids. Her health history also included cataract removal in her mid‐50s, tinnitus, brittle teeth, and a heart murmur.

The initial genetic evaluation was prompted by pain and numbness in the extremities at age 55 years. Imaging studies revealed spinal stenosis of T11‐12 and some spinal cord impingement treated with surgical decompression of the spinal cord. Her stature was 146.7 cm (≤3rd centile), weight 91.2 kg (>97th centile), and head circumference 56.4 cm (60th centile). The arms were disproportionately short with more severe shortness of the humeri. There were no ventral elbow creases. Her fingers were short and distally tapered. Short lower extremities and brachydactyly of the toes were also present. Dysmorphic facial features are noted in Figure 1.

Health concerns included glaucoma, hypertension, and hypercholesterolemia. She continued to have increasing symptoms of stiffness and pain involving her back, groin, and knees causing limitations of her mobility.

**Figure 2:**
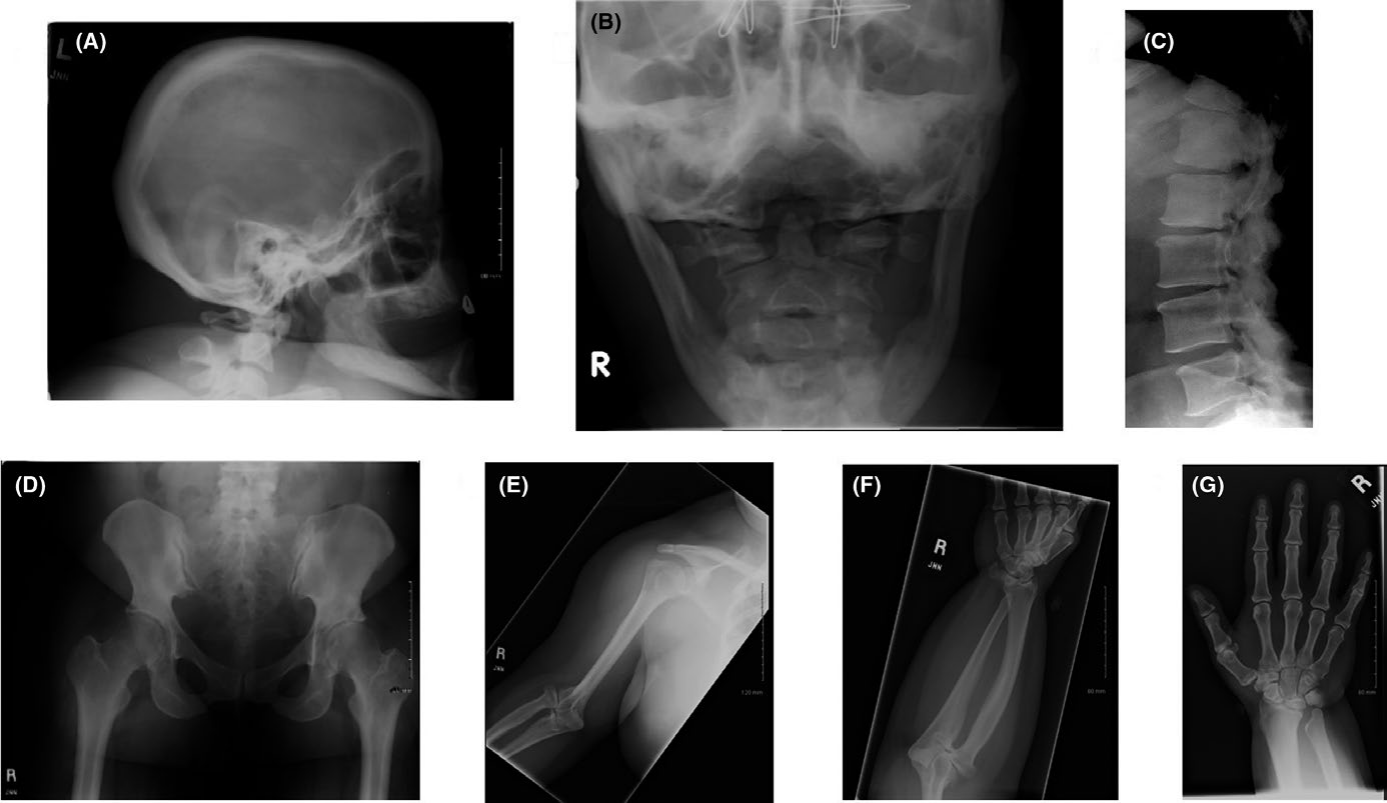
A, The calvaria is thickened. B, C1 is hypoplastic and the odontoid process of C2 is partially bifid. C, Spinal stenosis is noted. D, The iliac wings are hypoplastic and the femoral necks are in mild valgus position. E, The humerus is short and proximally expanded with a distorted distal end and subluxed radial head. F, There is marked mesomelic shortness due to a short, club‐shaped ulna with a bowed radius. An osseous bridge connects the proximal ends of radius and ulna. G, The metacarpals are mildly short and plump. The proximal carpal bones small and deformed.

Skeletal anomalies found during the first skeletal survey of Patient 2 at age 55 years were thought to be consistent with Robinow syndrome, as were her clinical features. However, this diagnosis was considered unlikely upon finding no molecular alteration by *ROR2* mutation screening. The radiographic findings are illustrated in Figure 2.

## 3 MOLECULAR FINDINGS

### 3.1 Patient 1

Sanger sequencing of the intronless *FZD2* gene (NM_001466.3) was performed on DNA extracted from blood under standard PCR conditions. The primers were designed using Primer Express 3.0 (Life Technologies Carlsbad, California, United States) with M13 tails added for sequencing. AmpliTaq Gold PCR master mix (Life Technologies) was used for PCR amplification under conditions as follows: 94°C for 5 minutes, followed by 35 cycles of 94°C during 30 seconds, 60°C for 30 seconds, and 72°C for 1 minute 19 seconds and finishing with 72°C for 5 minutes. The amplified product was purified with EXOSAP (Affymetrix Santa Clara, California, United States) and was sequenced using BigDye (Life Technologies) per manufacturer's instructions using a GeneAmp PCR System 9700 Thermal Cycles (Applied Biosystems Foster City, California, United States). Capillary electrophoresis was performed on an Applied Biosystems 3730xl sequencer (Life Technologies). A heterozygous G>A change was identified at nucleotide 1644 (c.1644G>A), expected to introduce a premature stop codon instead of the normally encoded tryptophan at position 548 of the FRIZZLED2 protein (p.Trp548*). This alteration was previously described with functional studies revealing dysregulation of the WNT pathway.^2^ Targeted analysis of the clinically unaffected mother and daughter was negative. The patient's father was not available for testing but was reported to be unaffected.

### 3.2 Patient 2

Analysis of the *ROR2* gene performed in 2009 failed to confirm the hypothesis of Robinow syndrome. Chromosomal microarray (Affymetrix Genome‐wide SNP 6.0) and singleton whole exome sequencing (WES) on an investigational basis were negative.

Exome sequencing in 2016 using standard laboratory procedures^6^ identified a heterozygous nucleotide alteration c.1301G>T, which results in a glycine being replaced by a valine (p.Gly434Val) in the FRIZZLED2 protein. This alteration was likely not reported in the prior investigational WES due to the association of the *FZD2* gene and autosomal dominant omodysplasia not yet being reported. Sanger sequencing of the intronless *FZD2* gene (NM_001466.3) was performed on DNA extracted from blood under standard PCR conditions confirmed the NGS finding. The patient's clinically unaffected mother and brother did not carry the alteration of interest. The patient's father was not available for testing but was reported to be unaffected.

## 4 DISCUSSION

Presented are two patients with alterations in the *FZD2* gene. Patient 1 has a phenotype consistent with autosomal dominant omodysplasia, while Patient 2 has features reminiscent of Robinow syndrome. Phenotypic similarity of the two disorders has been previously reported.^4^, ^7^ Patient 1 has a heterozygous p.Trp548* alteration, which has been previously reported in an affected mother and child.^2^ Patient 2 carries a missense alteration p.Gly434Val, which is predicted by several predictive algorithms to be pathogenic. The p.Gly434Val alteration has been recently described in a patient with OMOD2 and parental testing in that case indicated that the alteration appeared de novo.^3^ These findings suggest that pathogenic alterations in the *FZD2* gene may be recurrent.

Our second patient has a mild intellectual disability, which is not a feature reported in other patients. Additionally, she requires ongoing treatment for seizures, also noted in the patient described by Nagasaki et al^4^ who required anticonvulsant medication for afebrile convulsions in early childhood.

Previous studies have shown that FRIZZLED2 is broadly expressed in the developing head and limb buds in several animal model systems.^2^ The nucleotide alteration in Patient 1 and in the patients reported by Saal et al^2^ results in truncation of the FRIZZLED2 protein, which has been shown to impact Wnt signaling.

The missense alteration (p.Gly434Val) identified in Patient 2 is located in the fifth transmembrane domain of the FRIZZLED2 protein. It is possible this alteration impacts localization of this protein to the plasma membrane, though further studies will need to be pursued to show the specific effect.

The clinical effects of either a premature stop codon or a missense alteration in the *FZD2* gene appear to be rather similar, as both patients described here display many common physical features. Although one exception is the inadequate midline fusion in the facial region of Patient 2, prior reports also described hypertelorism in addition to cleft lip and cleft palate associated with alterations in the *FZD2* gene.^2^, ^3^ Differences in genomic background may contribute to the severity or differences in the clinical features that are seen.

In summary, we identified alterations in the *FZD2* gene in a previously described patient and a previously undescribed patient. This report broadens the phenotypic knowledge of OMOD2 and confirms a previously reported Robinow syndrome‐like phenotype.

## CONFLICT OF INTEREST

None declared.

## AUTHOR CONTRIBUTIONS

HEW: was the primary manuscript author. RJL and MJF: provided molecular analysis and reviewed the manuscript. JLF, JGL, JWS, SAS, and NLC: involved in patient evaluation and provided review of the manuscript.
